# Long-Lasting Maternal Depression and Child Growth at 4 Years of Age: A Cohort Study

**DOI:** 10.1016/j.jpeds.2010.03.008

**Published:** 2010-09

**Authors:** Iná S. Santos, Alicia Matijasevich, Marlos Rodrigues Domingues, Aluísio J.D. Barros, Fernando C.F. Barros

**Affiliations:** Post-graduate Program in Epidemiology, Federal University of Pelotas, Pelotas, Brazil

**Keywords:** BMI, Body mass index, EPDS, Edinburgh Postnatal Depression Scale, GA, Gestational age, H/A, Height-for-age, LBW, Low birth weight, LMP, Last menstrual period, W/A, Weight-for-age, W/H, Weight-for-height, WHO, World Health Organization

## Abstract

**Objective:**

To investigate the association between sustained maternal depression at 12, 24, and 48 months post-partum and child anthropometry at age of 4 years.

**Study design:**

A total of 99.2% of the 4287 children born in 2004 in Pelotas, Brazil, were enrolled in a cohort study. At 3, 12, 24, and 48 months, mothers were interviewed and provided information on several characteristics. Maternal depression was investigated through the Edinburgh Postnatal Depression Scale (EPDS). Weight-for-age, height-for-age, and weight-for-height z-scores at 48 months, according to World Health Organization growth curves, were the outcomes. Multivariate analyses were conducted through logistic regression.

**Results:**

At the 48-month follow-up, of the 3792 children, prevalence of underweight was 1.7%; stunting, 3.6%; wasting, 0.6%; and overweight, 12.2%. Depression (EPDS ≥13) was observed in 17.9% of the 3748 mothers. Of the mothers, 4.7% were persistently depressed at the 12-, 24-, and 48-month visits. In crude analyses, maternal depression was positively associated with underweight and stunting. After adjustment, maternal depression was not associated with any of the anthropometric indices.

**Conclusion:**

Long-lasting maternal depression at 12, 24, and 48 months post-partum is not a risk factor for impaired child growth or overweight at age of 4 years.

See editorial, p 355

Poor maternal mental health has become the subject of attention and concern because of the high rates of depression in mothers with small children. A meta-analysis of studies from developed countries that assessed women for depression during pregnancy or the first year post-partum showed that as many as 19.2% of women have a depressive episode during the first 3 months post-partum, most of these episodes having onset after delivery.^1^ Investigations undertaken in resource-poor countries found very high rates of depression, generally 2- to 3-times higher than those observed in industrialized countries.^2-4^ Maternal depression adversely affects women's health and well-being, with symptoms such as depressed mood, tiredness, insomnia, low self-esteem, and a lack of energy and interest in the environment. Maternal depression has been investigated as a risk factor for impaired caretaking capacity to provide sensitive, responsive, and stimulating care, which is important for infants' and children's psychological development, intellectual competence, psychosocial functioning, rate of psychiatric morbidity, and physical well-being.^5,6^

Child growth is a process with a multi-factorial determination, involving genetic and environmental factors (food availability, feeding practices, health, and morbidity, besides general child care) that act by promoting or restricting the individual inherent potential for growth. Post-natal maternal depression may contribute to the risk of growth impairment and illness through several ways, including early cessation of breastfeeding and inadvertent reduced maternal attention to and care of children's needs.^7,8^

This study aimed to investigate whether 48-month-old children of mothers with long-lasting depression (from 12-48 months post-partum) were at increased risk of impaired growth compared with children of non-depressed mothers. It was hypothesized that long-lasting maternal depression at 12, 24, and 48 months postnatal would be significantly associated with wasting, underweight or overweight, and stunting at 4 years of age.

## Methods

Pelotas in Southern Brazil has a population of approximately 340 000 inhabitants, 93% of whom live in the urban area (2000 Brazilian Demographic Census). From Jan 1, 2004, to Dec 31, 2004, a birth cohort attempted to enroll all births from mothers who lived in the urban area of the city. Births were detected with daily visits to the 5 maternity hospitals, in which 99% of the births take place. Mothers were interviewed, and their newborns were examined within 24 hours of delivery.^9^ With a pre-tested structured questionnaire, detailed information was obtained on several maternal characteristics: demographic (age at delivery, skin color), socioeconomic (family income in the month before delivery, education, presence of a partner), smoking (≥1 cigarette/day in any trimester), pre-pregnancy weight, reproductive history, and health care use. Children were visited at home at 3, 12, 24, and 48 months. Losses and refusals to participate in the study were 4.3%, 5.7%, 6.5%, and 8.0% for each of the follow-up visits, respectively.

Underweight, stunting, and wasting at 48 months (the outcomes) were defined as weight-for-age (W/A), height-for-age (H/A), and weight-for-height (W/H) z-scores less than -2 SD; and overweight was defined as W/A z-score greater than +2 SD, calculated according to the World Health Organization (WHO) growth curves^10^ by using Anthro software (http://www.who.int/childgrowth/software/en/). Children were weighed alone and, whenever possible, undressed. When the mother did not allow the child to be totally undressed, any clothing was registered, and the weight was subsequently deducted from the measured weight. Tanita electronic scales (Tanita, Tokyo, Japan) with a 150-kg maximum and 100-g precision were used. Scales were calibrated weekly with standard weights. Height was measured with portable infantometers (1-mm precision) custom built for the study.

Repeated measures of maternal depression were obtained at 12, 24, and 48 months follow-up with the Edinburgh Postnatal Depression Scale (EPDS),^11^ a scale which expresses the intensity of depressive symptoms for the preceding 7 days. The validity of EPDS has been confirmed in the cohort.^12^ The EPDS was translated into Portuguese and back-translated into English. Three months after delivery, EPDS was administered to 378 mothers. As many as 15 days later, mothers were re-interviewed by mental healthcare professionals using a semi-structured interview on the basis of *International Statistical Classification of Diseases, 10th revision* Research Diagnostic Criteria, taken as the gold standard. An EPDS score ≥13 showed a specificity rate of 88.3% (83.9-91.9) and sensitivity rate of 59.6% (49.5-69.1) for depression diagnosis.^12^

Instead of the self-administered format, questions were posed to mothers as a single block in the same order as in the original instrument, within the cohort's regular interviews. Administration of EPDS as an interview is accepted by the instrument's authors^11^ and has been used previously.^12,13^ Maternal depression was categorized as: never depressed (EPDS <13 at all 3 follow-up visits), depressed in 1 or 2 follow-up visits, and always depressed (EPDS ≥13 at each follow-up visit).

Gestational age (GA) was estimated with an algorithm^14^ on basis of last menstrual period (LMP) whenever consistent with birth weight, length, and head circumference.^15^ When the LMP-based GA was unknown or inconsistent, the Dubowitz method (which was performed on almost all newborns) was adopted.^16^ Preterm birth was defined as birth <37 weeks of GA and low birth weight (LBW) was defined as <2500 g. Births with unknown GA accounted for 0.3% (n = 13) of births. Pre-pregnancy body mass index (BMI) was categorized according to the WHO classification.^17^ At each follow-up, information on the child's breastfeeding duration and hospital admissions were asserted with maternal report.

χ^2^ tests were used to investigate the association between each anthropometric index and maternal and child's characteristics. Logistic regression analysis was used to investigate the magnitude of the association between the outcomes and maternal depression. In the adjusted analyses, maternal depression was considered a proximal determinant of child's anthropometric outcomes, with effects that could be confounded by distal variables. An operational definition of confounding was used; that is, variables that were associated with both the outcome and the predictor of interest, and not part of the causal chain.^18^ All independent variables were tested as potential confounders, and only variables at the 0.20 significance level were entered in the adjusted analysis.^19^ All analyses were performed with Stata software version 10 (StataCorp LP, College Station, Texas). The study protocol was approved at each follow-up by the Medical Research Ethics Committee of the Federal University of Pelotas Medical School, affiliated with the Brazilian Federal Medical Council, and by the WHO. Written informed consent was obtained from mothers before any data collection.

## Results

The data collection of the cohort started in 2004 including 4287 children born in Pelotas (Brazil) by the time of the perinatal interview.^20^ By the 48-month follow-up, anthropometric information was collected from 3792 children and maternal depression was evaluated in 3748 mothers. At the age of 48 months, the prevalence of underweight was 1.7%; stunting, 3.6%; wasting, 0.6%; and overweight, 12.2%. Maternal depression at 48 months was observed in 17.9% of mothers. Longitudinal data analysis, from the 12-, 24-, and 48-month visits, revealed that 69.9% of the mothers were never depressed, 25.4% were depressed at 1 or 2 visits, and 4.7% presented long-lasting depression (at the 3 visits).

The distribution of anthropometric indices according to maternal characteristics is shown on Table I. Family income and maternal schooling were associated to children's growth on a linear fashion. Prevalence of underweight and stunting was higher in children of mothers with non-white skin color. Prevalence of stunting in children of adolescent mothers was more than twice as high as in children of mothers ≥34 years old. The higher the maternal parity, the higher the prevalence of underweight and stunting. Pre-pregnancy maternal BMI was inversely associated to underweight, stunting, and wasting. Children from mothers who smoked during pregnancy had a prevalence of stunting twice as high as that observed in children from non-smoking mothers.

Family income, maternal schooling, and maternal pre-pregnancy BMI were directly associated with children's overweight, and maternal parity was inversely related (Table I). Prevalence of overweight was higher in children of non-smoking mothers than in children of mothers who smoked during pregnancy.

Maternal depression history was positively associated with underweight and stunting (Table I). Prevalence of underweight in children whose mothers had long-lasting depression was 3 times higher than the prevalence in children from never-depressed mothers (3.6% and 1.2%, respectively). The same was observed in relation to stunting: prevalence in children from mothers with long-lasting depression was 5.9% compared with 3.1% in children from mothers who were never depressed. Maternal depression was not associated with child's overweight.

Mean W/A, H/A, and W/H z-scores in children from mothers who were never depressed were 0.465 (±1.227), -0.0829 (±1.048), and 0.740 (±1.174), respectively. For children of mothers depressed at 1 or 2 visits, the correspondent mean z-scores were 0.252 (± 1.220), -0.271 (± 1.101), and 0.624 (± 1.160), and for children whose mothers presented long-lasting depression, the values were 0.227 (±1.242), -0.325 (±1.066), and 0.693 (±1.284), respectively. For all the anthropometric indices, means according to the 3-exposure status were statistically different.

LBW and preterm birth were associated with increased prevalence of underweight, stunting, and wasting at 4 years of age (Table II). Being hospitalized during the first year of life resulted in a 2- to 3-fold higher proportion of growth deficits. Highest prevalence of wasting was observed in children who were never breastfed. LBW or preterm birth decreased the probability of being overweight at the age of 4 years.

The adjusted analyses focused on the relation between maternal depression and anthropometric indices at 48 months (Table III). After adjustment, maternal depression was not associated with any of the investigated anthropometric indices (Table III). Interaction between maternal depression and family income was tested with the Mantel-Haenszel test, and the result was statistically non-significant. Despite the lack of association, the highest odds ratios for underweight, wasting, and overweight were observed in children from mothers with long-lasting depression.

When anthropometric outcomes were analyzed as continuous variables, the effect of long-lasting maternal depression was statistically significant in crude analyses: -0.239 (±0.098) points in W/A (*P* < .001), -0.242 (±0.085) in H/A (*P* < .001), and -0.047 (±0.094) in W/H z-score (*P* = .04). After allowing for family income, maternal schooling, and parity, the association lost statistical significance: -0.046 (±0.097) for W/A z-score (*P* = .2), -0.049 (±0.083) for H/A z-score (*P=* .4), and 0.059 (0.094) for W/H z-score (*P* = .5).

To compare only mothers from the extreme categories of the exposure, post hoc analyses were conducted to estimate the effect of being a child from a mother with EPDS ≥13 in all 3 visits against being a child of a mother with EPDS <5 (n= 575) in all 3 visits. The observed odds for being exposed to a chronically depressed mother were 3.0-, 2.0-, 2.0-, and 1.3-times higher for children with underweight, stunting, wasting, and overweight than the odds for children from mothers with EPDS <5 in all 3 visits (very similar to the odds observed in the analyses aforementioned).

## Discussion

We found that at 4 years of age, the prevalence of stunting and overweight in children from the 2004 Pelotas Birth Cohort were, respectively, 1.4- and 5.0-times higher than the expected for a healthy population (2.5%). A deficit in linear growth and overweight had already been identified when these children were 12 months old.^21^ Since then, the prevalence of stunting decreased from 6.0% to 3.6% (a reduction of approximately 40%) and, on the contrary, the prevalence of overweight increased approximately 50% (from 8.2% to 12.2%). These findings are consistent with earlier publications, which showed that during the nutritional transition the Latin American countries are presenting one of the most important reductions in growth deficits,^22^ with the highest prevalence of overweight in children <5 years of age.^10^

Throughout the first 4 years of life of these children, nearly one-third of their mothers had been identified at least 1 time as depressed (30.1%), and almost one-quarter of them had long-lasting depression. Prevalence of underweight and stunting were, respectively, 3- and 2-times higher in children of mothers with long-lasting depression than in children of mothers who were never depressed. Adjusted analyses, however, showed that they are problems independent of maternal depression.

Some of the strengths of this study come first from its being a population-based birth cohort and from the identification of the cases being made with strictly anthropometric criteria, making the presence of any important selection bias unlikely. Second, definition of long-lasting maternal depression was based on results of 3 follow-up visits 1 to 2 years apart, aiming to identify mothers with chronic depression and to distinguish them from mothers with depression of shorter duration. The 3 categories of maternal depression allowed that, when present, a dose-response effect was detected. Third, this study involved large numbers of individuals with the outcome or exposed to maternal depression, so it seems unlikely that an association of any magnitude has been missed.

In the last decade, several studies exploring the association between maternal depression and child growth were published in the scientific literature. Six of the publications were from prospective cohort studies,^3,23-27^ in which the effect of maternal depression was adjusted for several potential confounders through restriction or multivariate analyses. Of those studies, 3 reported statistically significant association between maternal depression and impaired child growth.^3,23,24^ As has been suggested,^26^ reasons for differences in the results of these cohorts may be related to socioeconomic and sociocultural factors that may interact in determining the effect of maternal mental health on child nutrition. In an environment like in Asia, for example, in which women face great adversities and are less empowered, a depressed mother may be at high disadvantage to ensure appropriate nutrition for her child.^7^

Another reason for differences in studies results may be the strategy used for selection of potential confounders. Maldonado and Greenland,^28^ in a study to compare the performance of several strategies for helping to decide whether one should adjust to a variable, concluded that the significance test strategies perform best when the alpha level is set much higher than conventional levels (0.20). In this study, post hoc analyses were carried out to include in the final model only variables associated with the outcome below the .05 level of significance (data not shown). In such models, maternal depression remained significantly associated with child underweight. This result, however, may be caused by residual confounding, which may also explain some of the other studies in which significant effects persisted after adjustment.

In Brazil, 2 studies previously were conducted to test the association between maternal depression and growth impairment or overweight. The first was a small case-control study (60 cases : 45 controls) conducted in 2 primary health care units of a low-income housing area of Sao Paulo.^29^ The adjusted odds ratio for having a depressed mother was 2.6-fold higher in the cases compared with the controls. The second was a cross-sectional study including 589 mother-child dyads from low-income urban communities in Teresina, Northeast Brazil. Multivariate analyses showed that inadequate child growth from 6 to 24 months of age was sensitive to maternal social support, but not to maternal depression.^30^ Subsequent analyses showed that children of mothers with high depressive symptoms had 80% greater odds of short stature^31^ and 1.7- and 2.3-times higher odds of being greater than the W/H z-score cutoff points for the 85th and 95th percentile of the WHO growth curves, respectively.^32^

A finding common to most of the studies is that in crude analyses a positive association between maternal depression and child impaired growth was found. Comparability between findings of different studies, however, may be difficult because of sorts of methodological and contextual issues. For instance, the presence of maternal depression has been assessed through the use of different instruments, with variable accuracy, and administered at different pre-natal or post-natal periods; and, in cohort studies, duration of follow-up varied from 2 to 18 months of children's life. Moreover, any other study has reported the relation between long-lasting maternal depression and child growth until 4 years of age.

Maternal depression can easily and reliably be detected with several simple screening and diagnostic tests suitable to use in poor settings in which growth impairment is most prevalent. If there is a risk factor for growth failure or overweight, management of maternal depression would become one of the most promissory strategies for public health policies. Although this is not the case in our setting, the high prevalence of maternal depression 12 to 48 months after delivery highlights the need of a more integrated approach to maternal and child care at the community level. Health care workers need to be prepared to screen and recognize maternal depression and to treat it appropriately.

## Figures and Tables

**Table I:** Anthropometric indices at 48 months of age according to the maternal characteristics

Variables	n	Underweight %	Stunting %	Wasting %	Overweight %
***Family income (quintiles)***		*P < .001*	*P < .001*	*P = .021*	*P = .001*
1st	762	3.4	6.7	1.3	8.7
2nd	762	2.5	5.3	0.5	10.6
3rd	752	1.5	3.1	0.5	12.6
4th	791	0.4	2.0	0.3	14.1
5th	732	0.7	1.1	0.1	15.2
***Marital status***		*P = .767*	*P = .362*	*P = .688*	*P = .182*
Single	600	1.8	3.0	0.7	10.6
With partner	3199	1.7	3.8	0.5	12.5
***Skin color***		*P = .053*	*P = .007*	*P = .509*	*P = .060*
Black/other	1021	2.4	5.0	0.7	10.6
White	2778	1.4	3.1	0.5	12.8
***Age (years)***		*P = .304*	*P = .014*	*P = .484*	*P = .061*
<20	710	2.3	5.2	0.7	9.8
20-34	2566	1.6	3.5	0.4	12.6
>34	521	1.2	2.1	0.8	13.8
***Schooling (years)***		*P < .001*	*P = .001*	*P = .044*	*P = .001*
0-4	570	4.1	9.1	1.2	8.0
5-8	1566	2.0	3.7	0.6	10.6
9-11	1253	0.8	2.0	0.3	15.4
≥12	373	-	0.3	-	14.9
***Parity***		*P = .026*	*P = .001*	*P = .408*	*P < .001*
0	1491	1.1	2.8	0.4	15.3
1	1009	1.7	2.9	0.5	10.9
≥2	1298	2.4	5.2	0.8	9.7
***Pre-pregnancy BMI***		*P = .005*	*P = .036*	*P = .069*	*P < .001*
<18.5	166	4.2	5.4	1.8	3.0
18.5-24.9	2169	1.9	4.1	0.6	10.4
25.0-29.9	822	1.0	3.1	0.3	15.6
≥30	389	0.5	1.6	0.3	20.4
***Smoking in pregnancy***		*P = .112*	*P < .001*	*P = .880*	*P = .012*
Yes	1033	2.2	6.1	0.6	10.0
No	2766	1.5	2.7	0.6	13.0
***EPDS ≥13***		*P = .002*	*P = .021*	*P = .496*	*P = .314*
Never	2490	1.2	3.1	0.6	12.7
1-2 times	905	2.6	4.8	0.4	10.9
Always (3 times)	169	3.6	5.9	1.2	13.7

**Table II:** Anthropometric indices at 48 months of age according to the child characteristics

Variables	n	Underweight %	Stunting %	Wasting %	Overweight %
***Sex***		*P = .582*	*P =.636*	*P = .631*	*P = .977*
Male	1973	1.6	3.5	0.6	12.2
Female	1826	1.8	3.8	0.5	12.2
***Multiple birth***		*P =.545*	*P = .608*	*P = .387*	*P = .869*
Yes	78	2.6	2.6	1.3	12.8
No	3721	1.7	3.7	0.5	12.2
***LBW***		*P < .001*	*P < .001*	*P < .001*	*P = .001*
Yes	341	6.8	10.6	3.5	6.5
No	3457	1.2	3.0	0.3	12.8
***Preterm birth***		*P < .001*	*P < .001*	*P < .001*	*P = .011*
Yes	524	3.7	7.3	1.9	8.8
No	3270	1.4	3.0	0.3	12.7
***Hospitalization 1st year life***		*P < .001*	*P < .001*	*P = .024*	*P = .253*
Yes	701	3.3	6.7	1.2	11.0
No	3001	1.3	2.9	0.4	12.6
***Duration BF (months)***		*P = .772*	*P = .941*	*P = .010*	*P = .286*
Never	101	3.0	4.0	3.0	8.0
0.1-3	1236	1.8	3.7	0.7	12.4
3.1-6	543	1.3	3.1	0.4	13.4
6.1-12	678	1.5	3.4	0.2	13.7
>12	1233	1.8	3.9	0.6	11.2

*BF,* Breastfeeding.

**Table III:** Adjusted odds ratios for anthropometric indices at 48 months of age, according to maternal depression status (EPDS ≥13)

	EPDS ≥ 13			
Anthropometric indices	Never	At 1-2 follow-ups OR (95% CI)	At 3 follow-ups OR (95% CI)	*P*
Underweight	1.0	1.5 (0.8-2.8)	1.7 (0.6-4.8)	.319∗
Stunting	1.0	1.0 (0.6-1.5)	0.9 (0.4-2.0)	.934†
Wasting	1.0	0.6 (0.2-2.0)	1.5 (0.4-7.6)	.580‡
Overweight	1.0	1.0 (0.8-1.3)	1.6 (1.0-2.5)	.210§

*OR,* Odds ratio.

∗ Adjusted for family income, maternal skin color, maternal schooling, parity, pre-pregnancy BMI, smoking during pregnancy, preterm birth, hospitalization in the first year of life, and duration of breastfeeding.

† Adjusted for family income, maternal skin color, maternal schooling, age, parity, pre-pregnancy BMI, smoking during pregnancy, preterm birth, and hospitalization in the first year of life.

‡ Adjusted for family income, maternal schooling, pre-pregnancy BMI, preterm birth, hospitalization in the first year of life, and duration of breastfeeding.

§ Adjusted for family income, maternal skin color, marital status, maternal age, maternal schooling, parity, pre-pregnancy BMI, smoking during pregnancy, and preterm birth.
